# Expression of Collagen XIII in Tissues of the Thyroid and Orbit With Relevance to Thyroid-Associated Ophthalmopathy

**DOI:** 10.1167/iovs.65.4.6

**Published:** 2024-04-02

**Authors:** Oula Norman, Tuuli Vornanen, Hanna Franssila, Johanna Liinamaa, Elina Karvonen, Tommi Kotkavaara, Vesa-Matti Pohjanen, Ritva Ylikärppä, Taina Pihlajaniemi, Merja Hurskainen, Anne Heikkinen

**Affiliations:** 1 ECM-Hypoxia Research Unit, Faculty of Biochemistry and Molecular Medicine, University of Oulu, Oulu, Finland; 2 Department of General Surgery, Oulu University Hospital, and Medical Research Centre, University of Oulu, and Oulu University Hospital, Oulu, Finland; 3 Department of Ophthalmology, Oulu University Hospital, and Research Unit of Clinical Medicine, Medical Research Centre, University of Oulu, and Oulu University Hospital, Oulu, Finland; 4 Cancer and Translational Medicine Research Unit, Medical Research Centre Oulu, University of Oulu, and Oulu University Hospital, Oulu, Finland

**Keywords:** collagen XIII, thyroid-associated ophthalmopathy, autoantibody, extraocular muscle, thyroid

## Abstract

**Purpose:**

Antibodies against collagen XIII have previously been identified in patients with active thyroid-associated ophthalmopathy (TAO). Although collagen XIII expression has been described in extraocular muscles and orbital fat, its detailed localization in extraocular and thyroid tissues and the connection to autoimmunity for collagen XIII remain unclear. Our objective was to map the potential targets for these antibodies in the tissues of the orbit and thyroid.

**Methods:**

We evaluated the expression of collagen XIII in human patient and mouse orbital and thyroid tissues with immunostainings and RT-qPCR using *Col13a1^−^^/^^−^* mice as negative controls. *COL13A1* expression in Graves’ disease and goiter thyroid samples was compared with *TGF-β1* and *TNF*, and these were also studied in human thyroid epithelial cells and fibroblasts.

**Results:**

Collagen XIII expression was found in the neuromuscular and myotendinous junctions of extraocular muscles, blood vessels of orbital connective tissue and fat and the thyroid, and in the thyroid epithelium. Thyroid expression was also seen in germinal centers in Graves’ disease and in neoplastic epithelium. The expression of *COL13A1* in goiter samples correlated with levels of *TGF-B1*. Upregulation of *COL13A1* was reproduced in thyroid epithelial cells treated with TGF-β1.

**Conclusions:**

We mapped the expression of collagen XIII to various locations in the orbit, demonstrated its expression in the pathologies of the Graves’ disease thyroid and confirmed the relationship between collagen XIII and TGF-β1. Altogether, these data add to our understanding of the targets of anti-collagen XIII autoantibodies in TAO.

The pathogenesis of thyroid-associated ophthalmopathy (TAO), characterized by inflammation of extraocular muscle, orbital fat, and connective tissue, has puzzled researchers ever since it was first described almost two centuries ago. Nowadays it is a common autoimmune disorder that affects between 20% to 50% of patients with Graves’ hyperthyroidism, but the current treatment options are often suboptimal because of inability to target the pathogenetic mechanisms of the disease.^1^^,^^2^ At its worst, the condition is sight-threatening for the patient, and in such cases aggressive treatments such as decompressive surgery can have serious complications, including strabismus, diplopia, and optic nerve damage.^3^

The current theories regarding TAO pathogenesis center around autoimmunity against thyroid-stimulating hormone receptor (TSHR), which is commonly recognized as the cause of Graves’ disease but is also expressed in orbital tissues.^4^^,^^5^ Also implicated in the process is insulin-like growth factor 1 receptor (IGF-1R) activation, potentially as a result of dimerization with TSHR, as highlighted by the approval of teprotumumab, an anti-IGF-1R antibody, for the treatment of TAO.^6^ Teprotumumab has proved to be an effective treatment and is currently recommended as a first-line therapy in cases where proptosis or diplopia is present, but long-term efficacy and safety data, as well as availability outside of the United States, are still lacking.^7^ The target cells of both types of autoantibody inside the orbit are various subtypes of fibroblast and blood-derived fibrocyte that react by producing inflammatory cytokines and hyaluronan, thus contributing to the characteristic edema and inflammation in the orbit.^4^^,^^5^ Questions like why only a fraction of Graves’ disease patients develop eye symptoms or how ophthalmopathy is sometimes associated with Hashimoto's thyroiditis nevertheless remain unanswered. Smoking, thyroid dysfunction, previous radioactive iodine treatment, high levels of serum TSHR antibodies, and hypercholesterolemia have been reported to increase the risk of occurrence or progression of TAO.^8^ Novel therapies (e.g., an anti-CD40 monoclonal antibody [iscalimab] and TSHR autoantibody [K1-70]) are under investigation. Moreover, correcting hypercholesterolemia has been shown to benefit the patients with TAO.^8^

Alongside the prevailing TSH receptor autoimmunity hypothesis, antibodies against collagen XIII have been implicated as a component in the pathogenesis of thyroid-associated ophthalmopathy and as a marker of active ophthalmopathy.^9^^,^^10^ Anti-collagen XIII autoantibodies have previously been reported in myasthenia gravis, and a deficiency of collagen XIII, a postsynaptic component of the neuromuscular junction (NMJ), has been shown to result in congenital myasthenic syndrome type 19.^11^^–^^14^ Although multiple localizations and functions have been suggested for collagen XIII expression in orbital tissues,^15^^–^^17^ the exact consequences of autoimmunity against collagen XIII in TAO remain unclear. To clarify these issues, we present here data on the expression of collagen XIII protein and mRNA in different tissues of the orbit and thyroid.

## Material and Methods

### Mouse Maintenance and Experimentation

Collagen XIII knockout mice (*Col13a1*^−^^/^^−^; B6.129-Col13a1^tm3.1Pih^/Oulu; MGI:4838409)^13^ were maintained in a specific pathogen-free facility under an internal license (03/2016) at the University of Oulu Laboratory Animal Center. The mice have been created by deleting the 5′ regulatory sequences and the first protein-coding exon and thus do not express any collagen XIII.^13^ Mouse work was performed in compliance with the European Community Council Directive (September 22, 2010; 2010/63/EEC), national legislation and regulations for the use of laboratory animals and the ARVO Statement for the Use of Animals in Ophthalmic and Vision Research. The animals were housed at 21°C with a 12-hour light/dark cycle and fed Teklad global 18% protein rodent diet with food and water as desired. Adult littermate mice of both sexes from heterozygous breedings were used.

### Human Patient Samples

This research was completed in accordance with the Declaration of Helsinki as revised in 2013 and national legislation. Written informed consent was obtained from all the participants, and approval was obtained from the Ethical Committee of the Northern Ostrobothnia Hospital District in Oulu (DNo 31/2015, amendment 6/2016, amendment 1/2019). The thyroid samples were obtained from patients undergoing thyroidectomy for goiter or Graves’ disease at Oulu University Hospital (Table). The extraocular muscle samples were from patients undergoing eye muscle resection for correction of strabismus. Samples from the orbicularis oculi muscle and the underlying adipose tissue were from eyelid surgery. Thus all the samples represented patient tissue material that would otherwise have been discarded. The first samples were collected in September 2015. One goiter patient had hyperthyroidism and received treatment for it but tested negative for TSHR antibodies. Orbital radiotherapy or other immunosuppressants were not used for the treatment of TAO in these patients, but one goiter patient was treated with methotrexate and adalimumab for psoriatic arthritis.

**Table.** Characteristics of Thyroid Sample Donors

	Goiter (N = 18)	Graves’ Disease (N = 9)
Patient information		
Age range (years), median	26–77, 54	30–77, 46
Previous/Current smokers	8/4	5/1
TAO symptoms	N/A	5
Prior therapy for hyperthyroidism or TAO		
Antithyroid drugs	1	9
Radioactive iodine	1	2
Glucocorticoids	0	2

### Immunohistochemistry

Human thyroid formalin-fixed paraffin-embedded sections were deparaffinized with xylene and rehydrated by incubation in descending concentrations of ethanol. Epitope retrieval consisted of boiling the slides in 10 mM Tris- 1 mM EDTA, pH 9, for 15 minutes. The samples were then stained using the EnVision kit (Agilent, Santa Clara, CA, USA) with anti-collagen XIII antibody (1:500, HPA050392; Atlas Antibodies, Bromma, Sweden) according to the kit instructions. Hematoxylin was used as the counterstain. The primary antibody was omitted from a negative control, and consecutive sections were stained with hematoxylin and eosin for correlation. The slides were scanned with a Leica slide scanner (Leica, Wetzlar, Germany).

### Cell Culture Experiments

CI-huThyrEC human thyroid epithelial cells were purchased from InSCREENeX (cat. no. INS-CI-1017; Braunschweig, Germany). Primary human thyroid fibroblasts were purchased from ScienCell Research Laboratories (cat. no. 3730; Carlsbad, CA, USA). All the cells were grown according to suppliers’ instructions using the recommended media and other reagents (huThyrEC medium, cat. no. INS-ME-1017; InSCREENeX), epithelial cell coating solution (cat. no. INS-SU-1020; InSCREENeX), and fibroblast medium (cat. no. 2301; ScienCell Research Laboratories). Human recombinant TNF was purchased from PeproTech, Inc. (Cranberry, NJ, USA) and used at 25 ng/mL for the experiments. Human recombinant TGF-β1 was obtained from PeproTech and used at 10 ng/mL. In one set-up, thyroid epithelial cells were also cultured on depleted medium which consisted of huThyrEC basal medium with 1% fetal bovine serum, amphotericin B, penicillin, and streptomycin. The cells were seeded onto six-well plates and cultured until they reached a sufficient confluence. Medium containing TNF or TGF-β1 was added, and after the duration of the experiment the cells were lysed with the Tri reagent system (Sigma-Aldrich Corp., St. Louis, MO, USA). The experiments were repeated three times (N = 3) with two technical replicates per experiment, using the average of the two as the final value for the experiment.

### Whole-Mount Immunofluorescent Staining

The mouse samples were dissected and fixed in 4% PFA/PBS for 10 minutes at room temterature (RT), and the human thyroid samples and extraocular muscle samples were fixed with 2% to 4% PFA/PBS for five to 10 minutes. The samples were then permeabilized with 1% Triton X-100/PBS overnight at 4°C and neutralized with 100 mM glycine/PBS for 15 minutes at RT, followed by incubation in blocking solution (2.5% goat serum, 2.5% BSA, 0.2% Triton X-100) for a minimum of 30 minutes at 4°C. The following rabbit anti-collagen XIII antibodies and dilutions were used: HPA050392, Atlas Antibodies (1:100), affinity-purified anti-human NC3 in-house antibody (1:100),^18^ and anti-mouse NC3 in-house antibody (1:100).^19^ Details of other antibodies are presented in Supplementary Table S1. Samples were incubated with primary antibodies diluted in the blocking buffer overnight at 4°C, washed three times with PBS, and incubated with secondary antibodies supplemented with 4′,6-diamidino-2-phenylindole (DAPI) that was used for staining the nuclei for one hour at RT. The samples were washed as previously before mounting with Immu-mount (Shandon, Fisher Scientific, Waltham, MA, USA) and imaged with a Zeiss Cell Observer spinning disk confocal microscope (Zeiss, Oberkochen, Germany) and Olympus Fluoview 1000 (Olympus, Tokyo, Japan) and Zeiss LSM 700 laser scanning confocal microscopes (Zeiss).

### Immunofluorescent Staining on Cryo Sections

Cryo-sections (5 µm) of frozen tissues embedded in Tissue-Tek O.C.T. (Sakura Finetek USA, Inc., Torrance, CA, USA) compound were fixed in 4% PFA/PBS for 10 minutes at RT. The samples were then permeabilized with 1% Triton X-100/PBS for 10 minutes at RT and neutralized with 100 mM glycine/PBS for 15 minutes at RT, followed by incubation in a blocking solution (2.5% goat serum, 2.5% BSA, 0.2% Triton X-100) for an hour at RT. Sections were incubated with the following anti-collagen XIII antiserum dilutions in the blocking solution overnight: anti-human NC3 in-house antiserum (1:500)^18^ and anti-mouse NC3 in-house antiserum (1:800).^19^ Details of other antibodies are presented in Supplementary Table S1. Samples were washed three times with PBS and incubated with secondary antibodies supplemented with DAPI used for staining the nuclei for one hour at RT. After PBS washes the samples were mounted with Immu-mount (Shandon) and imaged with a Leica Stellaris confocal microscope (Leica). Coverslips were detached after imaging with overnight incubation of PBS, and sections were stained with hematoxylin and eosin. The slides were scanned with a Hamamatsu slide scanner (Hamamatsu Photonics, Hamamatsu, Japan).

### Correlative Light and Electron Microscopy

Paraffin-embedded slides were stained with immunofluorescence and imaged with a Leica SP8 Falcon confocal with Lightning deconvolution. Coverslips were detached in PBS and slides were postfixed with 1% osmium tetroxide and embedded in Epon LX112 (Ladd Research Industries Inc., Williston, VT, USA) using gelatin capsules. Thin sections were post-stained and imaged using a Tecnai G2 Spirit 120 kV transmission electron microscope (Thermo Fisher Scientific, Waltham, MA, USA) with a Quemesa CCD camera (Media System Lab Srl, Rovereto, Italy). The resulting images were correlated with the eC-CLEM^20^ plugin for Icy.^21^

### Tissue Transcript and Protein Level Analyses

RNA and protein were isolated from thyroid tissue samples and cells with the Tri reagent system (Sigma-Aldrich) according to the manufacturer's instructions. A few random RNA samples were run on the Agilent Bioanalyzer 2100 to ensure adequate quality. For the tissue samples, 20 µg of RNA were further purified with the RNEasy micro kit (Qiagen, Hilden, Germany) according to the kit instructions to remove residual PCR inhibitors. Reverse transcription was performed with the iScript cDNA synthesis kit (Bio-Rad Life Science, Hercules, CA, USA). RT-qPCR reactions were run on a CFX96 system (Bio-Rad Life Science) using the iTaq Universal SYBR Green Supermix kit (Bio-Rad Life Science), as previously described.^22^ *HPRT1, ACTB* and *SDHA* were chosen as reference genes for the tissue samples, based on a previous report,^23^ whereas *HPRT1* and *ACTB* were used for the cells. Primer sequence and efficiency information can be found in Supplementary Table S2.

### Mouse Thyroid Hormone Measurements

Blood was collected from the inferior vena cava of 25-week-old female mice at termination and allowed to coagulate for two hours at RT. Serum was collected after 2000 rpm centrifugation with an Eppendorf microcentrifuge for 20 minutes at RT. Serum free thyroxine (S-T4-V) measurements were performed by Nordlab, Oulu, Finland.

### Statistical Analysis

The statistical analyses were performed with GraphPad Prism 5 (GraphPad Software, San Diego, CA, USA). Linear regression was used to examine the correlation between *TGFB1* and *COL13A1* expression. Student's *t*-test was used to compare means in the serum thyroxine measurements.

## Results

### Collagen XIII Is Located at the Myotendinous and Neuromuscular Junctions in the Extraocular Muscle and Affects NMJ Integrity

**Figure 1.**
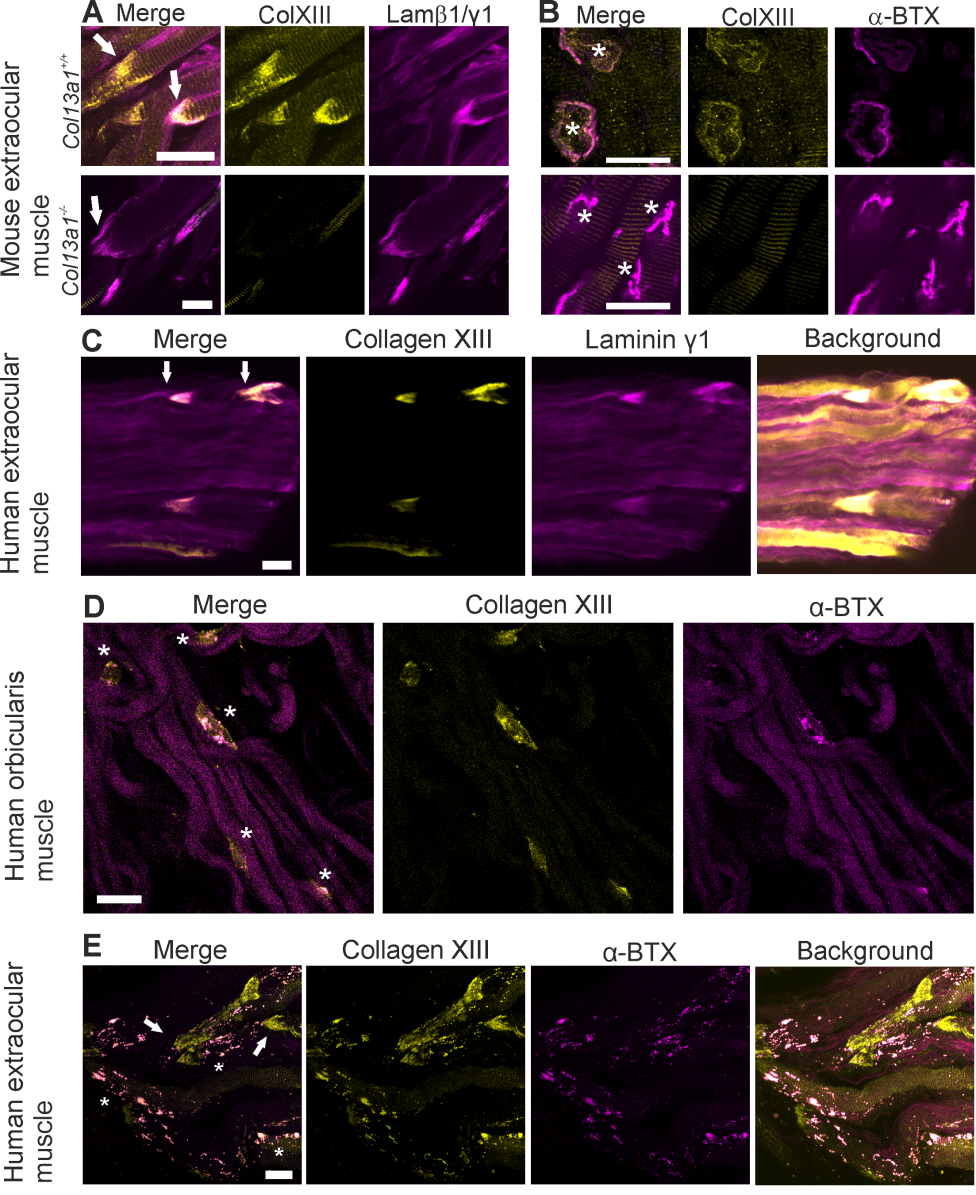
Collagen XIII expression patterns in human and mouse extraocular muscles. (**A**, **B**) Whole mount immunofluorescent stainings of *Col13a1^+/+^* (wild-type) and *Col13a^−^^/^^−^* (knockout) mouse rectus muscle identifying myotendinous (**A**), and neuromuscular junctions (**B**) labeled with antibodies against collagen XIII (*yellow*) and laminin β1 (*Col13a1^+/+^*; A; *magenta*) or laminin γ1 (*Col13a^−^^/^^−^*; A; *magenta*), or with α-BTX (B; *magenta*). (**C**–**E**) Whole mount immunofluorescent stainings of human samples labeled with antibodies against collagen XIII (*yellow*) and laminin γ1 (C; *magenta*) or with α-BTX (**D**, **E**; *magenta*), showing collagen XIII expression in the human extraocular muscle MTJ (**C**), in NMJs of human orbicularis oculi muscle (**D**) and in MTJs and *en grappe* NMJs at the distal end of a human rectus muscle (**E**). MTJs marked with arrows, NMJs marked with asterisks. Scalebars are 10 µm in A-B and 20 µm in C-E. The images are maximum intensity projections of confocal stacks.

To better understand the role of collagen XIII in TAO we first sought to determine the expression of collagen XIII in the tissues affected in TAO, namely the extraocular muscles, connective tissue, and extraocular fat. To investigate the expression pattern of collagen XIII in the extraocular muscles, we stained these muscles as taken from mice and also pieces of human extraocular muscle removed during strabismus surgery with antibodies against collagen XIII. In both the human and mouse samples collagen XIII was located in the myocyte plasma membrane of the myotendinous and neuromuscular junctions, as established previously by our group^12^^,^^13^^,^^24^^,^^25^ (Fig. 1). In the mouse myotendinous junction (MTJ) it was co-localized with laminin β1, an established component of MTJs,^26^ at the end of the muscle fiber in the wild-type (*Col13a1^+/+^*), whereas the knockout (*Col13a1^−^^/^^−^*) mouse samples were devoid of collagen XIII staining (Fig. 1A). In the human samples the staining was seen in a similar pattern at the distal end of the rectus muscle (Fig. 1C). In the NMJ collagen XIII was closely colocalized with the acetylcholine receptor clusters labeled with α-bungarotoxin (α-BTX). Its expression on mouse *en plaque* NMJs is demonstrated in Figure 1B and that in human orbicularis oculi muscle NMJs in Figure 1D. Collagen XIII expression on human *en grappe* NMJs and MTJs is shown in Figure 1E.

After confirming collagen XIII expression in the NMJs of the extraocular muscles, we sought to compare the extraocular muscle phenotype of the collagen XIII-knockout mice with previous observations of compromised NMJ integrity.^12^^,^^13^^,^^24^ The NMJs in the various extraocular muscles of knockout mice were found small and immature compared to those of the wild-type mice (Fig. 1B and Supplementary Fig. S1), but we could not detect differences in the severity of the phenotype between the muscles.

### Collagen XIII Associates With Blood Vessels in Orbital Tissue

**Figure 2.**
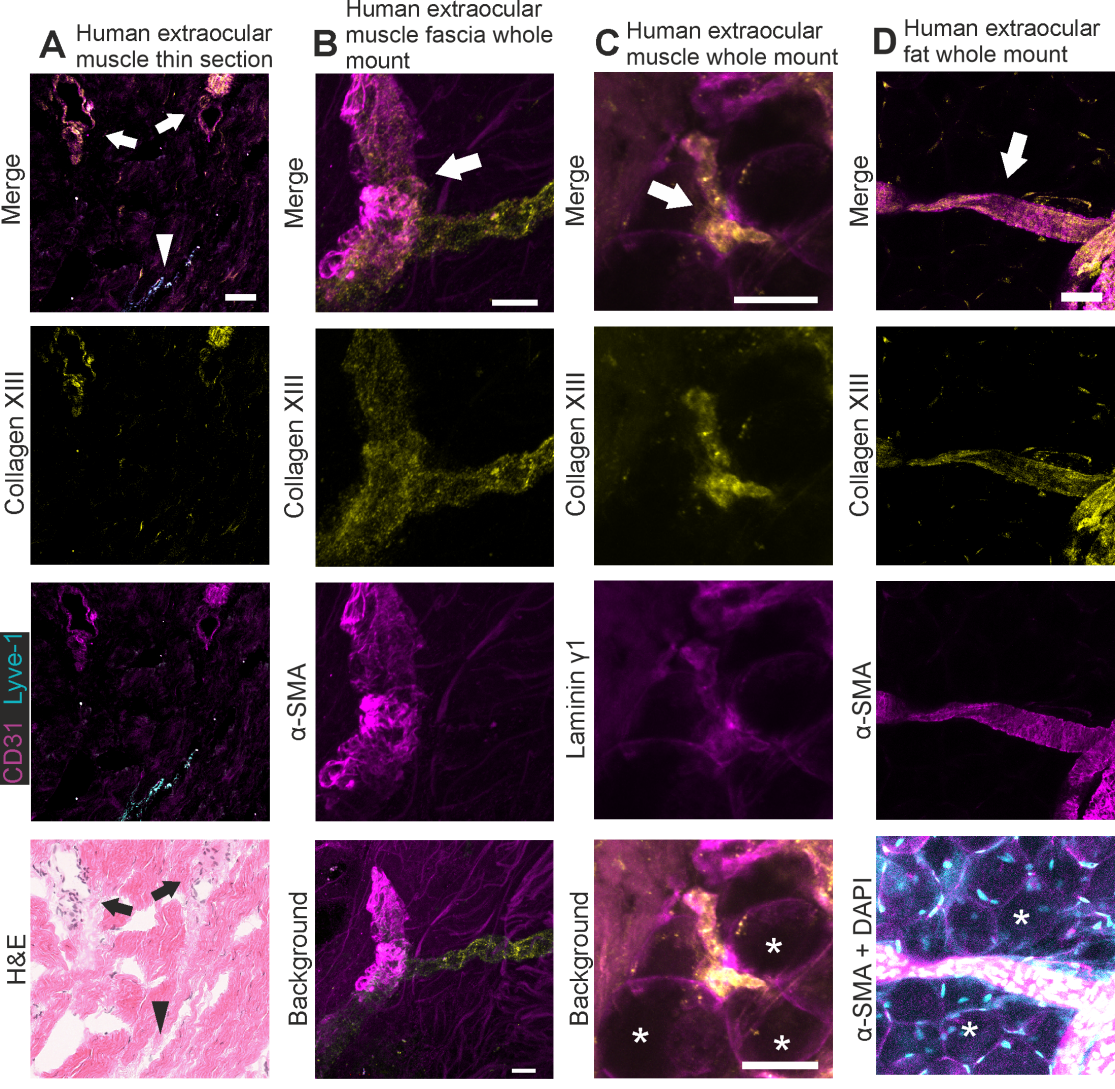
Collagen XIII expression in orbital blood vessels. (**A**) immunofluorescent labeling of a human cryo section with antibodies against collagen XIII (*yellow*), CD31 (*magenta*), and Lyve-1 (*cyan*), with hematoxylin-eosin (H&E) staining of the same section, showing collagen XIII expression in cross-sections of the blood vessels of human extraocular muscles. (**B**–**D**) Whole mount immunofluorescent labelings of human samples with antibodies against collagen XIII (*yellow*), α-SMA (**B**, **D**; *magenta*) and laminin γ1 (**C**; *magenta*), showing collagen XIII expression in the blood vessels of human extraocular muscles (**B**, **C**) and in extraocular fat (**D**). Blood vessels marked with *arrows*, adipocytes marked with *asterisks* and a lymphatic vessel marked with an *arrowhead*. *Scalebars* are 200 µm in **A**, 10 µm in **B**, and 50 µm in **C** and **D**. The images **B–D** are maximum intensity projections of confocal stacks. In the H&E image, white balance and brightness have been adjusted for easier viewing.

In addition to its expression in extraocular muscle NMJs and MTJs, collagen XIII staining is seen in vascular structures associated with extraocular muscles and fat (Fig. 2). As shown in a cross-section, collagen XIII is expressed in CD31-positive blood vessels but not in the Lyve-1-positive lymphatic vessels (Fig. 2A). In a whole mount-stained blood vessel of the muscle fascia (Fig. 2B), collagen XIII expression was observed in both α-smooth muscle actin-positive and -negative branches. A collagen XIII signal was also detectable in an intramuscular blood vessel associated with intramuscular fat (Fig. 2C), and a blood vessel in the orbital fat tissue was also positive for collagen XIII (Fig. 2D). Similar findings have been reported by Zainul et al.,^27^ who detected collagen XIII in the mouse perineural vasculature, and by the Human Protein Atlas (https://www.proteinatlas.org/ENSG00000197467-COL13A1/single+cell+type), which reports collagen XIII expression by vascular smooth muscle cells based on single-cell RNA sequencing.^28^

### Collagen XIII Is Expressed in Follicles and Blood Vessels of the Thyroid and Is Associated With Certain Pathologies

**Figure 3.**
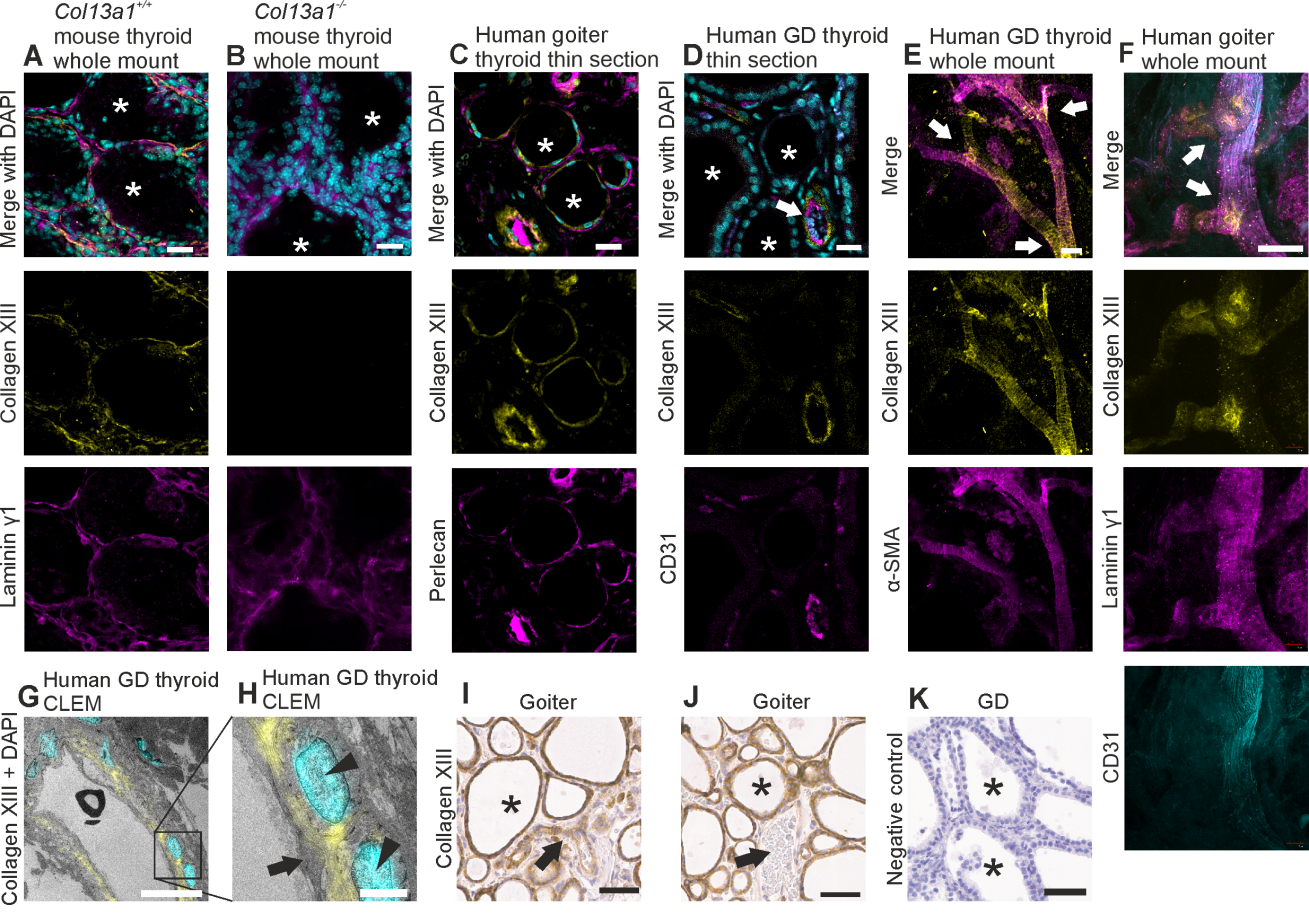
Collagen XIII expression in thyroid follicles and blood vessels. (**A**, **B**) Immunofluorescent labeling for collagen XIII expression (*yellow*) and laminin γ1 (*magenta*) with DAPI (*cyan*) to stain nuclei in *Col13a1*^+/+^ and *Col13a1*^−/−^ mouse thyroid whole mounts, showing collagen XIII expression in the basal surfaces/basement membranes of follicular cells in the wild-type (**A**). (**C–F**) Immunofluorescent labeling for collagen XIII expression (*yellow*) and CD31 (**D**, *magenta*; **F**, *cyan*) or perlecan (**C**, *magenta*) or α-SMA (**E**, *magenta*) together with DAPI (**C**, **D**, *cyan*) or laminin γ1 (**F**, *magenta*) in human thyroid samples (**C**, **D**, thin paraffin sections, **E**, **F**, whole mounts), showing collagen XIII expression in blood vessels (**D–F**) and selected follicles (**C**). GD, Graves’ disease. (**G, H**) CLEM of human thyroid samples, where collagen XIII is colored yellow and DAPI cyan, showing collagen XIII location in a blood vessel wall. (**I–K**) Immunohistochemical staining of collagen XIII in a goiter sample with (**I**) and without vessel signal (**J**), primary antibody omitted in negative control (**K**). Blood vessels marked with *arrows*, thyroid follicles marked with *asterisks*, and nuclei marked with *arrowheads*. *Scale bars*: 20 µm in **A–D, F**; 50 µm in **E, I–K**; 10 µm in **G**; and 2 µm in **H**. The images **E–F** are maximum intensity projections of confocal stacks.

**Figure 4.**
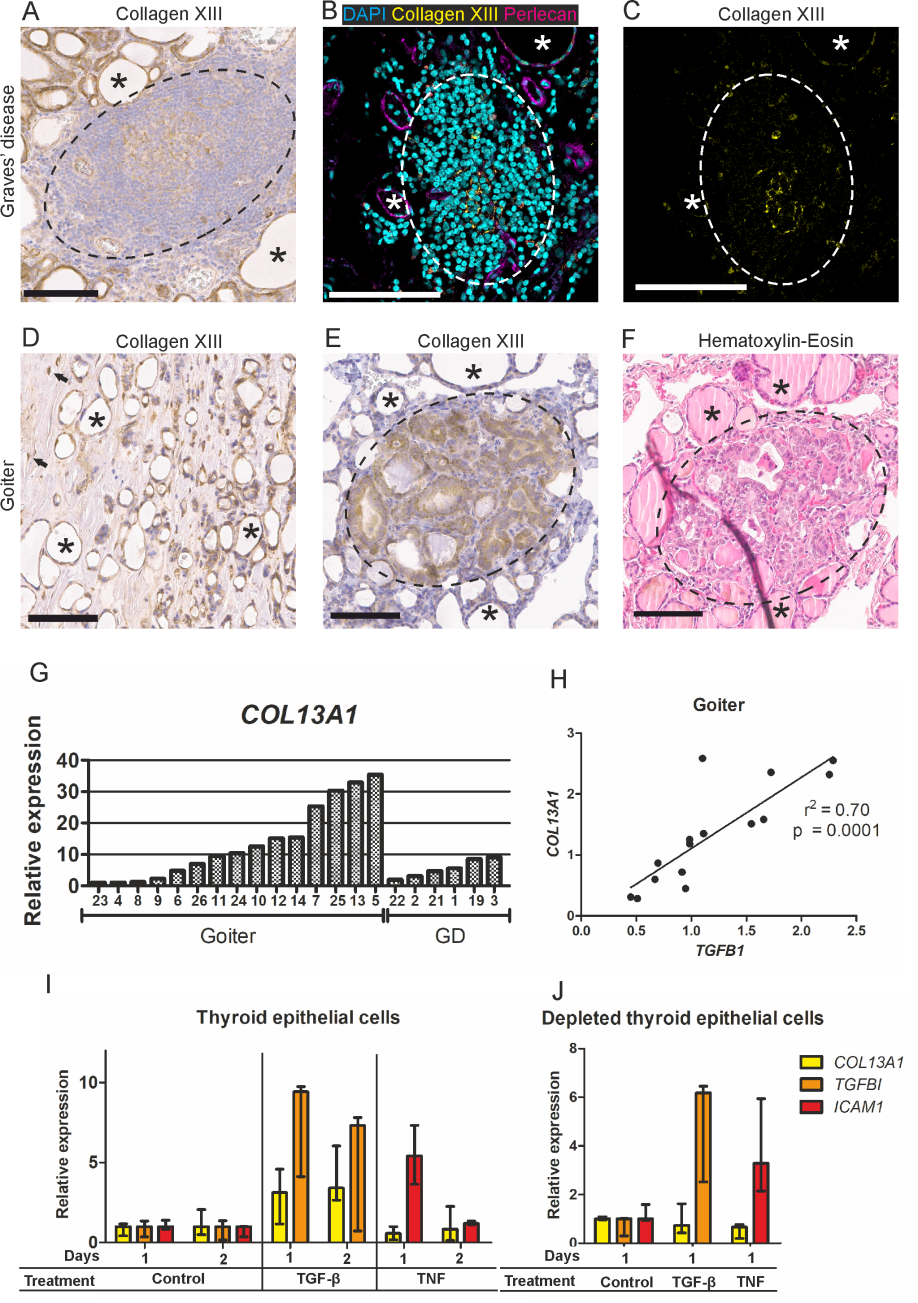
Collagen XIII in goiter and Graves’ disease thyroids. (**A**) Immunohistochemistry of a germinal center reaction in a Graves’ disease thyroid examined for collagen XIII. Germinal center marked with *dashed line*. (**B**, **C**) Immunofluorescence of a lymphocytic infiltrate in a Graves’ disease thyroid section examined for collagen XIII (*yellow*). Co-labeling with DAPI (*cyan*) and perlecan (*magenta*). Lymphocytic infiltrate marked with *dashed line* in both images. (**D**) Collagen XIII immunohistochemistry of connective tissue in a goiter thyroid, immunopositive fibroblasts indicated by *arrows*. (**E**) Collagen XIII immunohistochemistry of an incidental papillary microcarcinoma marked with *dashed line* in a goiter thyroid. (**F**) Hematoxylin-eosin staining of the corresponding area on an adjacent section. *Scalebars*: 100 µm. Thyroid follicles marked with *asterisks* in all images. (**G**) Expression of *COL13A1* in thyroid cohort samples detected by RT-qPCR. GD, Graves’ disease. (**H**) Correlation between *TGFB1* and *COL13A1* expression in goiter thyroids, linear regression. (**I**, **J**) Expression of *COL13A1* detected by RT-qPCR in human thyroid epithelial cells after treatment with TGF-β1 (positive control *TGFBI*) or TNF (positive control *ICAM-1*) either in growth (**I**) or depleted (**J**) medium (median with range, N = 3).

To study the involvement of collagen XIII in thyroid disease, we performed immunofluorescent and immunohistochemical staining on mouse thyroids and human donor samples from goiter and Graves’ disease patients undergoing thyroidectomy. In the immunofluorescently stained wild-type mouse thyroid whole mounts, collagen XIII was located in the basal surfaces/basement membranes of follicular cells, while the samples from knockout mice were negative (Figs. 3A, 3B). Although normally located in follicles, its knockout had no direct effect on the serum levels of free thyroxine in mice (Supplementary Fig. S2). In the immunofluorescently stained human thyroid samples, collagen XIII was detected in some, but not all, of the follicles (Figs. 3C, 3D). There were no clear differences in follicular staining between the goiter and Graves’ disease thyroids. As in the extraocular tissues (Fig. 2), collagen XIII was associated with selected blood vessels (Figs. 3D–F). Particularly vessel branching points were found intensively stained for collagen XIII in the whole mount staining (Figs. 3E, 3F). The collagen XIII staining pattern resembled that of smooth muscle cells in blood vessel walls in the whole mount staining (Fig. 3E). We then performed correlative light and electron microscopy (CLEM) to identify the collagen XIII-expressing structures and found collagen XIII to be located in the basement membrane between the vascular endothelium and the underlying smooth muscle cells (Figs. 3G, 3H). To assess the histopathology of goiter and Graves’ disease thyroids, we performed immunohistochemical staining for collagen XIII (Figs. 3I–K), resulting in intracellular staining of the follicular cells in both goiter (Figs. 3I, 3J) and Graves’ disease (Fig. 4A) samples. Similarly to the results gained with immunofluorescence, some blood vessels showed a collagen XIII signal (Fig. 3I) in immunohistochemistry while others were negative (Fig. 3J).

We focused next on the various pathologic features of Graves’ disease and goiter that were positive for collagen XIII in immunohistochemical staining. Diffuse collagen XIII staining was observed in germinal centers of the Graves’ disease samples (Fig. 4A), and a corresponding immunofluorescence signal was seen in lymphocytic infiltrates (Figs. 4B, 4C). In areas with connective tissue, some of the stromal cells in the goiter thyroids appeared to be collagen XIII-positive (Fig. 4D). Also, in one goiter thyroid an incidental papillary microcarcinoma, confirmed by hematoxylin-eosin staining of an adjacent section (Fig. 4F), showed increased collagen XIII staining (Fig. 4E) compared to the adjacent normal epithelium.

### Collagen XIII and TGF-β mRNA Levels Correlate in Goiter Thyroids

To assess possible quantitative differences in collagen XIII expression between cohorts, we performed an RT-qPCR analysis for *COL13A1* mRNA (Fig. 4G). The differences between the samples were relatively large in the goiter samples, whereas in Graves’ disease the intragroup variation was considerably smaller. Because collagen XIII has been shown to be upregulated by TGF-β,^29^ we investigated its expression and found a strong correlation with that of collagen XIII in the goiter samples (Fig. 4H) but not in Graves’ disease (not shown). We also tested *TNF* to see whether collagen XIII was connected with inflammation, but no such correlation was observed (not shown).

### Collagen XIII Expression in Thyroid Epithelial Cells Is Induced by TGF-β

To identify the potential cellular source of *COL13A1* upregulation, we treated human thyroid epithelial cells and fibroblasts with *TGF-β1* and TNF. Addition of *TGF-β1* to the growth medium induced *COL13A1* expression in the thyroid epithelial cells after 1d or 2d of treatment (Fig. 4I), but when the same cells were grown in depleted medium the addition of *TGF-β1* did not upregulate *COL13A1* (Fig. 4J)*.* TNF administration did not affect the expression of *COL13A1* (Figs. 4I, 4J). The positive controls for the treatments were upregulation of TGF-β induced (*TGFBI*) and intercellular adhesion molecule 1 (*ICAM1*).^30^^–^^32^ Thyroid fibroblasts did not upregulate *COL13A1* in response to either treatment when *TGFBI* and matrix metalloproteinase 9 (*MMP9*) were used as positive controls (Supplementary Fig. S3).

## Discussion

Collagen XIII in the extraocular muscles is evidently located at the NMJ and MTJ and in some blood vessels, thus resembling its expression in other skeletal muscles.^12^^,^^13^^,^^24^^,^^25^^,^^27^ The knockout mouse samples were entirely negative for collagen XIII staining, confirming the specificity of the antibody. In the NMJ, collagen XIII is an important component in the development of the NMJ^12^^–^^14^ while in the MTJ its role is still unclear. The NMJ localization and the mouse phenotype identified here are well in line with the reports of mild extraocular muscle myasthenia in some patients with congenital myasthenic syndrome type 19.^14^^,^^33^^–^^38^ Moreover, collagen XIII was also shown here to associate with CD31-positive blood vessels in the extraocular muscle, and, in the whole mount staining, signals were observed in some α-SMA–positive segments but also in α-SMA–negative branches in the extraocular muscle fascia and in the extraocular and intramuscular fat. Its expression at these locations provides a potential target for autoantibodies against collagen XIII and may help to explain the inflammatory reactions and dysfunction observed in the extraocular muscles in active TAO. Tentatively, autoantibodies could bind collagen XIII at these locations and trigger orbital inflammation and cellular damage through cell- and complement-mediated pathways.

The expression of collagen XIII in association with blood vessels has been demonstrated previously by different methods.^27^^,^^39^^,^^40^ Besides the blood vessels of the orbital tissues, intramuscular fat, muscle fascia, and extraocular fat, we demonstrated this expression in the blood vessels of human thyroid follicles imaged as whole mounts and with CLEM, where it was limited to the basement membrane between the endothelial and smooth muscle cells. This finding is supported by data from the Human Protein Atlas indicating that collagen XIII is expressed by smooth muscle cells, from which its ectodomain may be shed into the pericellular matrix. Different segments of blood vessels exhibited different amounts of collagen XIII staining, which may indicate that collagen XIII expression is a feature of one or multiple specialized vascular cell populations found in the various regions of the vascular tree.^41^ As a vascular wall basement membrane constituent, collagen XIII may, for example, contribute to vessel wall permeability or provision of extracellular cues to surrounding cells. Intense collagen XIII expression at the branching blood vessels gives room for speculation that collagen XIII could contribute to blood flow regulation which might be worth studying further.

It was also observed here that the expression patterns of collagen XIII in human thyroid samples varied to some extent between the immunofluorescently and immunohistochemically stained samples, even with the same patient and using the same antibody. Unlike the immunohistochemistry, not all follicles were positive for collagen XIII with immunofluorescence. In addition, the epithelial immunohistochemical signal appeared to be intracellular rather than plasma/basement membrane-associated, and lacked the typical features of normal collagen XIII signals. The RNA sequencing data from the Human Protein Atlas^28^ do not support intracellular expression of collagen XIII in follicular cells, but epithelial expression has been shown in pathological epithelial cells from cases of pulmonary fibrosis,^42^ bladder cancer^43^ and papillary thyroid cancer.^44^^,^^45^ Accordingly, an incidental papillary microcarcinoma in one of the present samples exhibited increased collagen XIII staining relative to the surrounding normal-looking cells in a goiter thyroid sample. Only a fraction of follicular basement membranes in the human samples were positive for collagen XIII, suggesting that the expression might be affected by disease-associated factors. In Graves’ disease collagen XIII expression was also detected in germinal centers formed in the lymphoid follicles of thyroids, and these may be considered hotspots of affinity maturation for immune cells.^46^ No sequencing information is available on anti-collagen XIII autoantibodies, but other types of pathological autoantibody contain evidence of somatic hypermutation following a germinal center response,^46^ suggesting that the expression observed in germinal centers may be important for the generation of the anti-collagen XIII autoantibodies. Because all the thyroid material used here was derived from diseased donors, the exact nature of collagen XIII expression in the intact, healthy thyroid still remains to be confirmed.

We investigated here the differences in collagen XIII expression observed between human thyroid samples and found induction in gene expression to correlate with *TGF-β1* but not with *TNF*, which we studied further by means of cell experiments. In line with previous results on collagen XIII^29^ and other ECM proteins,^47^ exposure to TGF-β1 upregulated collagen XIII production in epithelial cells, whereas TNF had no such effect. Under starved conditions this upregulation did not occur, suggesting a lack of one or more relevant interactor(s). Because collagen XIII expression responds to pathology-associated signals conveyed by at least TGF-β1, a proven actor in the pathophysiology of autoimmune thyroid diseases,^48^ such signals may promote its involvement in disease.

Altogether, we present information about the potential target tissues of the TAO-associated anti-collagen XIII autoantibodies. However, our study is limited by the fact that the Graves’ disease patients with TAO were treated before the thyroidectomy, which is supposed to lower the level of anti-collagen XIII antibodies,^10^ and thus their assessment was not pursued. Another limitation is that most of the orbital tissue donors did not have TAO as orbital surgery is rarely indicated in patients with active TAO. As such, the findings presented here offer a plausible connection to TAO but confirmation on whether the anti-collagen XIII autoantibodies are pathogenic or not requires still more investigation.

## Supplementary Material

Supplement 1
