# Supplementary material for: Expression of Collagen XIII in Tissues of the Thyroid and Orbit With Relevance to Thyroid-Associated Ophthalmopathy
Source: Invest Ophthalmol Vis Sci. 2024 Apr 2;65(4):6. doi: 10.1167/iovs.65.4.6 (PMC10996972; doi:10.1167/iovs.65.4.6)
Supplement: Supplement 1 [file iovs-65-4-6_s001.pdf]

Supplementary Table 1. Details of commercial antibodies used.

Primary antibodies		
Target	Dilution	Catalog number
Col13a1	1:100	HPA050392, Atlas Antibodies
CD31	1:100	550274, BD Biosciences
CD31	1:50	P2B1, DSHB, deposited by Wayner, E.A. / Vercellotti, G.
Lyve-1	1:100	MAB2125, R&D Systems
α-smooth muscle actin	1:100	A2547, Sigma-Aldrich
Laminin β1	1:100	MAB1928, Chemicon
Laminin γ1	1:100	MAB1914P, Chemicon
Secondary antibodies		
Alexa Fluor 405 goat anti-mouse	1:100	A31553, Molecular Probes, Invitrogen
Cy2 goat anti-rat	1:300	112-225-167, Jackson ImmunoResearch
Alexa Fluor 488 goat anti-rabbit	1:300	A11008, Molecular Probes, Invitrogen
Alexa Fluor 488 goat anti-rat	1:300	AB150157, Abcam
Cy3 goat anti-rabbit	1:300	111-165-144, Jackson ImmunoResearch
Alexa Fluor 594 goat anti-rat	1:300	A11007, Molecular Probes, Invitrogen
Alexa Fluor 594 donkey anti-rabbit	1:300	AB150076, Abcam
Alexa Fluor 647 goat anti-rabbit	1:300	111-605-144, Jackson ImmunoResearch
Alexa Fluor 647 goat anti-mouse	1:300	A21236, Invitrogen
Other		
4',6-diamino-2-phenylindole (DAPI)	1:300	
CF405S-conjugated α-bungarotoxin	1:300	00002, Biotium
Alexa Fluor 488-conjugated α-bungarotoxin	1:600	B13422, Molecular Probes, Invitrogen

Supplementary Table 2. Details of PCR primers used.

Gene	Forward primer sequence	Reverse primer sequence	Amplicon length	Efficiency %	R^2 of standard curve
ACTB	AGCCTCGCCTTTGCCG	CGCGGCGATATCATCATCCA	72	88.4	0.994
COL13A1	AGGCTGCTGGAACAAGTGAT	TGCATCATACTTCTGGCATTTTCA	116	107.9	0.989
HPRT1	CATTATGCTGAGGATTTGGAAAGG	CTTGAGCACACAGAGGGCTACA	129	107.4	0.991
ICAM1	AGCGGCTGACGTGTGCAGTAAT	TCTGAGACCTCTGGCTTCGTCA	115	95.6	0.993
MMP9	TTCTGCCCGGACCAAGGATA	ATGCCATTCACGTCGTCCTT	158	118.2	0.884
SDHA	TGTTGCAAACAGGAACCCGA	GTTCCCCAGAGCAGCATTGA	266	104.4	0.992
TGFB1	CGTGCTAATGGTGGAAAC	GCTCTGATGTGTTGAAGAAC	89	87.1	0.989
TGFBI	TGTGTGCTGAAGCCATCGTTG	CCGGCTTGTCTGAAAAGGTCA	250	89.0	0.991
TNF	GCTGCACTTTGGAGTGATCG	GCTTGAGGGTTTGCTACAACA	144	93.4	0.896

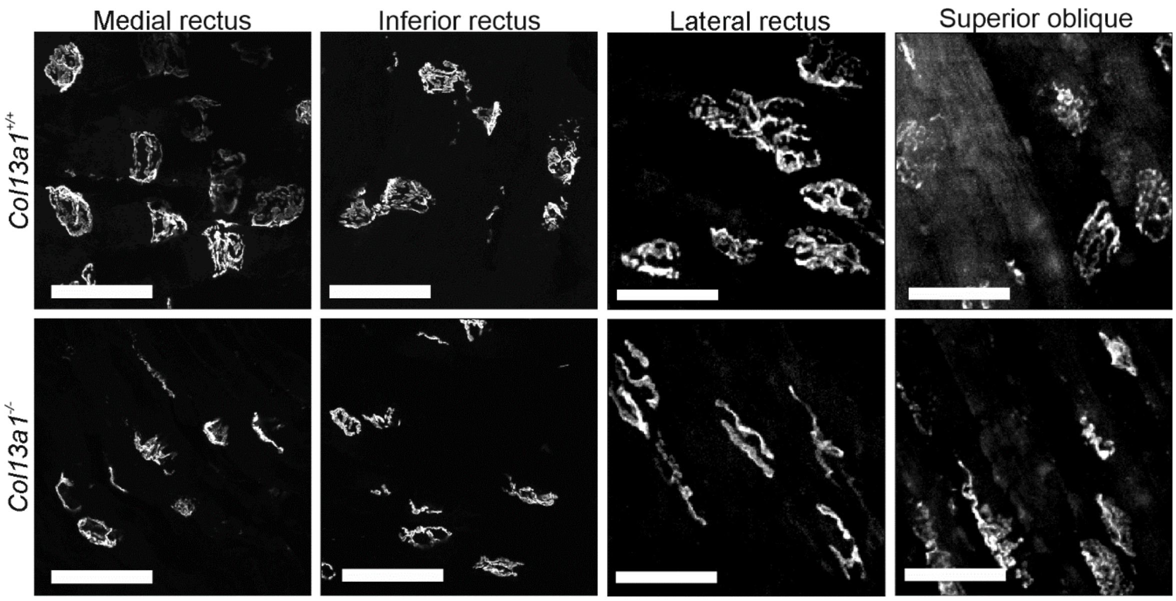

Supplementary Figure 1. NMJ morphology of extraocular muscles in mice. Acetylcholine receptor clusters are labeled with α-BTX in various adult *Col13a1*$^{+/+}$ and *Col13a1*$^{-/-}$ mouse oculomotor muscles. Scale bars 50 µm. Lateral rectus and superior oblique are from older mice and the NMJs are thus larger.

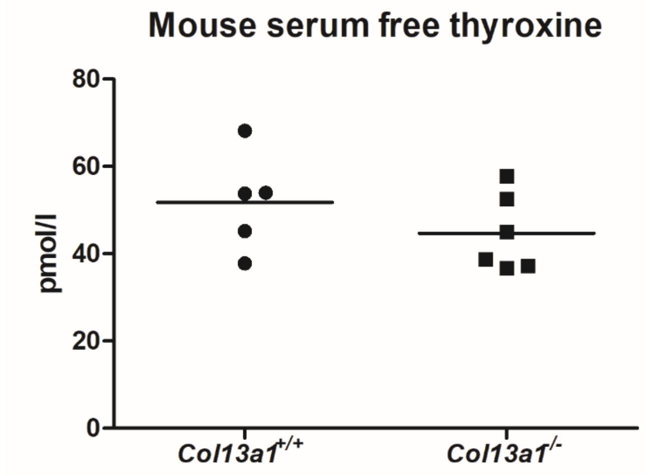

Supplemental Figure 2. Mouse serum free thyroxine. Clinical serum free thyroxine measurements from 25-week-old wild-type (N=5) and *Col13a1*$^{-/-}$ (N=6) female mice. P = 0.59. Student's t-test.

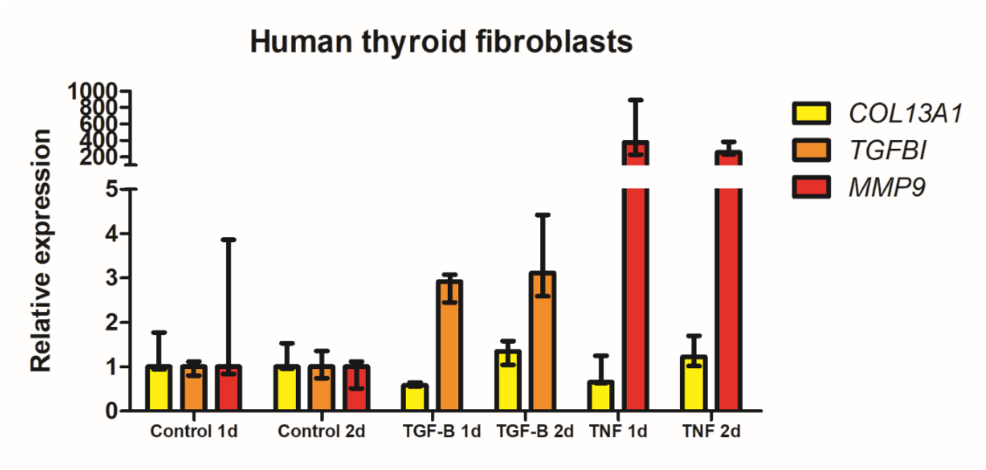

Supplemental Figure 3. Expression of *COL13A1* in human thyroid fibroblasts. RT-qPCR measurement of *COL13A1* expression in human thyroid fibroblasts after treatment with TGF-β (positive control *TGFBI*) or TNF (positive control *MMP9*) in growth medium. Shown median with range. N = 3.
